# Functioning in individuals with chronic fatigue syndrome: increased impairment with co-occurring multiple chemical sensitivity and fibromyalgia

**DOI:** 10.1186/1476-5918-6-6

**Published:** 2007-05-31

**Authors:** Molly M Brown, Leonard A Jason

**Affiliations:** 1 Department of Psychology, DePaul University, Center for Community Research, Chicago, IL, USA

## Abstract

**Background:**

Chronic fatigue syndrome (CFS), multiple chemical sensitivity (MCS), and fibromyalgia (FM) commonly co-occur. Some propose that CFS, MCS, and FM are manifestations of the same illness based on high rates of co-occurrence and overlapping diagnostic criteria. This study seeks to differentiate these diagnoses by comparing individuals with one or more illness on functioning, psychiatric comorbidity, coping style, and in vivo physical measures.

**Methods:**

Participants included 114 men and women who met criteria for CFS. FM was diagnosed during a physical examination, and MCS was assessed using a questionnaire. Participants were divided into four groups: CFS alone, CFS-MCS, CFS-FM, and CFS-MCS-FM. Self-report measures, a psychiatric interview, and in vivo physical measures were given.

**Results:**

43.9% met criteria for CFS alone, 23.7% met criteria for CFS-MCS, 15.8% met criteria for CFS-FM, and 16.7% met criteria for CFS-MCS-FM. The CFS-MCS-FM group was more disabled than the CFS alone group on measures of physical functioning, general health, and bodily pain. In vivo measures did not differ, but the CFS-MCS-FM group rated exertion higher than the CFS alone group.

**Conclusion:**

Individuals with CFS alone were the highest functioning group across several domains, such as disability, depression, and severity of symptoms. Participants with three diagnoses experienced the greatest amount of disability. While substantial co-occurrence of these illnesses was found, this study provides evidence that having more than one illness exacerbates one's disability beyond CFS alone.

## Background

Chronic Fatigue Syndrome (CFS) is a debilitating illness affecting approximately .42% of adults in the US [1]. A diagnosis of CFS based on the current US case definition [2] requires at least six months of persistent fatigue along with at least four of eight specified symptoms (e.g., impaired memory and concentration, unrefreshing sleep, post-exertional malaise). Relatively few patients with CFS completely recover from the illness [3], with a recovery rate of 0–6% and increased disability in 10–20% of patients over time [4].

Many of the symptoms associated with CFS are also characteristic of Fibromyalgia (FM) and Multiple Chemical Sensitivity (MCS). FM is a common rheumatologic ailment characterized by chronic generalized muscle pain, fatigue, and disrupted sleep. FM is present in about 3.4% of American women and .5% of men [5]. It has been found to occur following an acute medical illness, a traumatic injury, or surgery [6]. Proposed criteria for the diagnosis of FM require widespread muscular pain in conjunction with tenderness at a minimum number of tender points [7]. CFS and FM often co-occur [8-10]. It has been estimated that 20–70% of FM patients meet the criteria for CFS, and about 35–75% of patients with CFS also have FM [11-13]. Patients diagnosed with both CFS and FM have been found to be more disabled than those with either condition alone [14], suggesting that co-occurring CFS and FM has an additive effect on disability.

MCS is a commonly diagnosed illness, and is estimated to occur in 6% of adults in California [15]. MCS is defined as a chronic condition with reproducible symptoms involving multiple organ systems; with symptoms that are produced by low levels of exposure to multiple, chemically-unrelated substances and improve or resolve when the chemical agents are removed [16]. Common triggers include pesticides and perfumes [15], causing skin irritation, fatigue, fevers, and neurocognitive dysfunction. Treatment typically involves avoidance of exposure to offending chemicals using such tactics as eliminating carpeting, pesticides and cleaning agents from the home and avoiding other substances experienced as toxic [17]. Chemical avoidance was found to be an effective treatment in 93% of patients [18-20]. MCS is a commonly co-occurring illness with both CFS and FM. In a sample of 33 Gulf War veterans with CFS, 42% had concurrent MCS and 6% had concurrent FM [21]. Estimated rates of CFS comorbidity in persons with MCS range from 30% to 88% [11,22].

Based on the substantial co-occurrence of CFS, MCS, and FM, it is important to examine the functional status of individuals who experience two or more of these diagnoses. In a tertiary care sample, Ciccone and Natelson [23] found that women with all three diagnoses experienced poorer physical functioning, more pain, and more fatigue than those with CFS only. The study by Ciccone and Natelson is the only one to compare all three illnesses, but males were excluded from their sample. Additionally, that study did not examine stress, coping, quality of life or measures that did not rely on self-report data. The present study explored whether individuals with three illnesses are more disabled than those with two or one, and assessed different coping styles in response to their disability. Additionally, the use of in vivo physical measures was used to supplement information from self-report measures in order to employ multiple methods in gaining a more comprehensive understanding of this illness.

## Methods

### Participant recruitment

Participants were recruited from a variety of sources, including physician referrals. Information about the non-pharmacologic treatment trial study was disseminated to medical colleagues through mailings, phone communication, and invited grand rounds. In addition, study announcements for new participants were placed in local newspapers and recruitment offers were made at local CFS support group meetings. These efforts were continued until the target enrollment numbers were achieved. One hundred and fourteen individuals were recruited. All procedures were approved by the DePaul University Institutional Review Board. Informed consent was given by all participants.

Of the 114 individuals, 46% were referred by physicians, 34% were recruited by media (newspapers, TV, radio, etc.), and 20% stemmed from other sources (e.g., heard about the study from a friend, family member, person in the study, etc.). There were no significant demographic differences for patients recruited from these varying sources. Twenty-four additional individuals who were screened were excluded for various reasons (i.e., lifelong fatigue, less than 4 Fukuda symptoms, Body Mass Index > 45, melancholic depression or bipolar depression, alcohol or substance abuse disorder, autoimmune thyroiditis, cancer, lupus, rheumatoid arthritis). Approaches to reduce attrition included use of letters and telephone reminders of all appointments, flexibility regarding working around vacations and medical and other crises, reimbursement for transportation costs, and participant honoraria.

### Initial screening

All participants were required to be at least 18 years of age, not pregnant, able to read and speak English, and considered to be physically capable of attending the scheduled sessions. Patients who were bedridden or used wheelchairs were excluded due to the practical difficulties of making appointments. Referrals to local physicians who treat CFS and to support groups were offered to these individuals. After a consent form was filled out, prospective participants were initially screened by the third author, using a structured questionnaire.

### The CFS questionnaire

This screening scale was initially validated by Jason, Ropacki, et al. [24]. This scale is used to collect demographics, health status, medication usage, and symptom data. The CFS Questionnaire was revised, and administered it to three groups: those with CFS, Major Depressive Disorder, and healthy controls [25]. The revised instrument, which was used in the present study, evidences good test-retest reliability and has good sensitivity and specificity.

For each symptom, participants were asked to indicate if the symptom had been present for 6 months or longer, if the symptom began before the onset of their fatigue or health problems, and how often (never, seldom, often/usually, or always) the symptom is experienced. Participants were also asked to rate the severity of each symptom they endorsed on a scale of 0 to 100, where 0 = no problem and 100 = the worst problem possible. This is a numerical rating scale (NRS), which has been shown to be a consistently valid measure of symptom intensity, particularly for pain intensity [26]. The Fukuda et al. [2] case definition symptoms (i.e., impaired memory or concentration, sore throat, tender lymph nodes, muscle pain, multiple-joint pain, new headaches, unrefreshing sleep, and post-exertion malaise) were assessed.

A series of questions assessing MCS were included in the CFS Questionnaire. Questions that qualified a diagnosis of MCS included new awareness of odors, frequency of fever (not at all through daily), how sick one would be filling his or her own gas tank (not at all through a lot), and how sick one would be if he or she had to spend four hours in an enclosed shopping mall (not at all through a lot). These questions were derived from Donnay's [27] screening survey for CFS, MCS, and FM. This survey has evidenced diagnostic specificity of 96.7% and specificity of 98.3% (A. Donnay, personal communication, December 1, 2000).

### Structured clinical interview for DSM-IV

A semi-structured psychiatric interview was administered. The Structured Clinical Interview for DSM-IV (SCID) [28]. Axis I was used to establish psychiatric diagnoses. The professionally administered SCID allows for clinical judgment in the assignment of symptoms to psychiatric or medical categories, a crucial distinction in the assessment of symptoms that overlap between CFS and psychiatric disorders, e.g., fatigue, concentration difficulty, and sleep disturbance [3]. A psychodiagnostic study [29] validated the use of the SCID in a sample of CFS patients. Because CFS is a diagnosis of exclusion, prospective participants were screened for identifiable psychiatric and medical conditions that may explain CFS-like symptoms. These measures were completed at DePaul University and took approximately two hours. After the initial interview was completed, the patients' information was reviewed to ensure that they met all eligibility requirements. If an individual was eligible for the study, a medical appointment was set up. Conversely, if an individual was not eligible, alternative treatment options were discussed.

### Medical assessment of CFS

The physician screening evaluation included an in-depth medical and neurological history, as well as general and neurological physical examinations. A modified version of the CFS questionnaire was used to rule out other disorders [30]. Relevant medical information was gathered to exclude possible other medical causes of chronic fatigue, including exposure histories to tuberculosis, AIDS, and non-AIDS sexually transmitted diseases. Information on prescribed and illicit drug use was also assessed and recorded. The histories of all symptoms related to CFS were gathered. Laboratory tests in the battery were the minimum necessary to rule out other illnesses [2].

FM was diagnosed by the project physician during the medical assessment. The 1990 criteria from The American College of Rheumatology [7] were used. Participants received a diagnosis of FM if they had mild to severe tenderness in at least 11 out of 18 established tender point sites throughout the body.

### Medical Outcomes Study-Short Form-36

(MOS-SF-36). The MOS-SF-36, a 36 item broadly-based self-report measure of functional status related to health, identifies eight health concepts as perceived by the individual. The concepts include Physical Functioning, Role Functioning-Physical, Role Functioning-Emotional, Bodily Pain, General Health, Vitality, Mental Health, and Health Transition [31]. A higher score indicates better health or functioning. Test construction studies for the SF-36 [32,33] have shown adequate internal consistency, discriminate validity among subscales, and substantial differences between patient and non-patient populations in the pattern of scores. The SF-36 has also indicated sufficient psychometric properties as a measure of functional status in a CFS population [34].

### Fatigue Severity Scale (FSS)

Krupp, LaRocca, Muir-Nash, and Steinberg's [35] Fatigue Severity Scale was used to measure fatigue. This scale includes 9 items rated on 7-point scales and is sensitive to different gradations of fatigue severity. Most items in the Krupp fatigue scale are related to behavioral consequences of fatigue. Previous findings have demonstrated the utility of the Fatigue Severity Scale to discriminate between individuals with CFS, MS, and primary depression [36]. In addition, the Fatigue Severity Scale was normed on a sample of individuals with MS, SLE, and healthy controls. A study by Taylor, Jason and Torres [37] compared the Fatigue Scale [38] with the Fatigue Severity Scale [35] with a sample of healthy controls and a CFS-like group. Within a CFS-like group, the Fatigue Severity Scale was more closely associated with severity ratings for the eight Fukuda et al. [2] CFS symptoms as well as with functional outcomes related to fatigue.

### Beck Depression Inventory (BDI-II)

Because depression is the most commonly diagnosed psychiatric disorder in CFS [39], a quantitative measure of depression severity was used. Depressive symptomatology was measured with the BDI-II [40], a 21-item self-report instrument with well-established psychometric properties. This version of the BDI is more consonant with DSM-IV criteria for major depressive disorder. The BDI-II is the only depression rating scale to be empirically tested and interpreted for both depressed and non-depressed patients with CFS [41]. Also the Beck Depression Inventory has shown sensitivity to treatment changes in two cognitive behavioral treatment studies of CFS [42].

### Brief COPE

This inventory assesses how individuals cope with the stress in their lives [43]. It is derived from the Coping Orientation to Problems Experienced Scale [44], which consists of conceptually distinct problem-focused coping and conceptually distinct emotion-focused coping scales. This instrument has been validated and has adequate reliability. There are 28 items concerning ways of coping, and each is rated on a four point scales (anchor points ranging from not doing the coping strategy to doing it a lot). There are 14 coping methods found in these 28 items; the scales are as follows: Self-distraction, Active coping, Denial, Substance use, Use of emotional support, Use of instrumental support, Behavioral disengagement, Venting, Positive reframing, Planning, Humor, Acceptance, Religion, and Self-blame. There are two items for each of these 14 coping methods; the sum of the two items is the score for that particular coping method. Adequate psychometric properties of this instrument have been found [43].

### Brief Pain Inventory

The Brief Pain Inventory [45] was administered to measure the severity of pain and the interference of pain in the patient's life. Higher scores indicate more severe levels of persistent pain and higher levels of interference with functioning. This measure exhibits adequate levels of reliability to assess pain in noncancer samples, with coefficient alphas of .70 and above, also evidences good concurrent validity with other generic pain measures, and has been shown to be sensitive to changes in pain status over time [46].

### Pittsburg Sleep Quality Index

Sleep disturbances were examined by using the Pittsburg Sleep Quality Index, which was developed to measure sleep quality in psychiatric research [47]. This Index measures sleep disruptions and sleep quality. There are nineteen questions (on 0–3 scale) which generate seven "component" scores: subjective sleep quality, sleep latency, sleep duration, habitual sleep efficiency, sleep disturbances, use of sleeping medication, and daytime dysfunction. The sum of scores for these seven components yields one global score, which can range from 0 to 21, with higher scores indicating worse sleep quality.

### Actigraph

An actigraph is a small, light-weight, cost-efficient activity monitor that can be worn on the waist. It has a long battery life and can continuously collect data every minute of the day and night for 22 days before its memory is filled to capacity [48]. Unlike most activity monitoring devices, the actigraph is capable of recording movement intensity. The actigraph transduces activity using an accelerometer. An 8-bit analog-to-digital converter quantifies these measurements into 128 levels of positive acceleration and 128 levels of negative acceleration 10 times each second. Integration over the resulting sampling time of 0.1 s in combination with other details provided by Tryon and Williams [48] would result in measurement units of 1.664 milli-g/activity activity count. For simplicity, analog-to-digital (A/D) counts are retained as activity units. The average of 600 absolute A/D values is stored in memory at the end of every minute. Participants wore the actigraph on their waist at all times except for when bathing or sleeping for one week.

### Six minute walking test

As an in vivo measure of physical functioning, the six minute walking test [49] was used. The test measures the distance walked during a six minute interval. The test is a useful and reproducible measure of exercise tolerance, provides a simple practical guide to everyday disability, and does not require expensive apparatus [49].

### Rating of Perceived Exertion

The Rating of Perceived Exertion (RPE) measures perceived intensity of activity based on bodily sensations such as increased heart rate and breathing [50]. Higher scores indicate higher perceived exertion, with scores ranging from 6 (no exertion) to 20 (maximal exertion). The measure was given at 30 second intervals during the six minute walk test. The RPE has evidenced good reliability and validity, particularly for progressive exercise tests [51].

### Sit and reach

In order to gain an assessment of flexibility, we administered the sit and reach test, which is the most widely-used measure of flexibility and a primary component of most physical fitness tests. The test is designed to measure the extensibility of the hamstring muscles and the lower back articulations by evaluating the maximal reach an individual can make in a seated position. This test has excellent test-retest and intra-rater reliability [52].

### Hand grip

The hand dynamometer test was administered to measure grip strength in pounds, and is a good measure of loss of work capacity [53]. It is fast, easy to perform, and produces a reliable report that is simple to record [54].

### Employment status

This measure of role function consisted of work status (working vs. non-working). Employed included those who were working at least 20 hours per week or in school full-time. Unemployed included those who were retired, working fewer than 20 hours per week, not in school, or part-time student. We also assessed the number that are receiving disability benefits, the number on disability or temporary sick leave, and the number with work limitations due to the illness.

### Statistical analyses

Chi-square analyses were conducted between diagnostic groups (i.e., CFS only; CFS-MCS; CFS-FM; CFS-MCS-FM) and all categorical variables including race, gender, work status, marital status, whether or not a participant had children, and presence of psychiatric diagnoses. A series of one-way ANOVAs were performed for continuous variables of age, socioeconomic status, fatigue severity, and disability. To decrease the likelihood of a Type 1 error, Tukey HSD adjustments were made.

## Results

### Diagnostic status

Participants were categorized into four groups based on their diagnostic status of CFS alone; CFS and MCS (CFS-MCS); CFS and FM (CFS-FM); and CFS, MCS, and FM (CFS-MCS-FM). Of the 114 participants, 50 (43.9%) met criteria for CFS alone, 27 (23.7%) met criteria for CFS-MCS, 18 (15.8%) met criteria for CFS-FM, and 19 (16.7%) met criteria for CFS-MCS-FM.

### Sociodemographic characteristics

No significant differences were found across diagnostic categories (CFS alone; CFS-MCS; CFS-FM; and CFS-MCS- FM) for the sociodemographic variables of race, marital status, gender, age, socioeconomic status, or whether or not an individual had children. In regards to demographic characteristics, 16.7 % of the participants were male and 83.3% were female. The average age at baseline was 43.8 years. Regarding ethnicity, 87.7 % were Caucasian, 4.4 % were African-American, 4.4 % were Latino, and 3.5% were Asian-American. As for marital status, 49.1 % were married/living with someone, 33.3 % were single, and 17.6 % were either divorced or separated. In terms of work status, 24.6 % were on disability, 23.7 % were unemployed, 20.2 % were working part-time, 19.3 % were working full-time, 6.1% were retired, 4.4 % were part-time students, .9% were full-time students, and .9 % were working part-time and on disability. In terms of education, 47.4% had earned a standard college degree, 21.8% had a graduate or professional degree, 21.1% had partial college, and 9.7% had a high school/GED degree or less. Regarding psychiatric co-morbidity, 62.3% had a lifetime Axis 1 diagnosis, and 38.6% had a current Axis 1 diagnosis.

### Outcome measures

Whether individuals were working or not working was examined across the four groups using χ^2 ^analyses. There were marginally significant differences across the four groups, χ^2 ^(3, N = 114) = 7.73, p=.052. The CFS alone group had the highest percentage of working (54%), while the CFS-MCS- FM group had the lowest percentage of working (21.1%).

Outcome measures assessing fatigue severity, aspects of disability, pain, sleep, depression, and coping among the four diagnostic categories are presented in Table 1. An ANOVA was used to compare the four diagnostic groups on the eight scales of the MOS-SF-36. All post hoc tests described below used Tukey HSD. Overall significant differences were found for physical functioning (F (3, 108) = 4.40, p < .01), general health (F (3, 107) = 5.83, p < .001), social functioning (F (3, 107) = 4.27, p < .01), and bodily pain (F (3, 107) = 10.59, p < .001). Post hoc tests showed that the CFS-MCS-FM group was significantly more disabled than the CFS alone group on the physical functioning, general health, and bodily pain scales. Individuals with CFS-MCS-FM were also more disabled than individuals with CFS-MCS on the bodily pain scale. The former group revealed more disability on the general health scale than the CFS-FM group. There was a significant difference on the bodily pain scale between the CFS alone group and the CFS- FM group such that individuals with CFS- FM were more disabled by pain than individuals with CFS alone. Individuals with CFS-MCS were significantly more disabled with regards to social functioning than individuals with CFS alone.

**Table 1:** Outcome measures for individuals with CFS alone; CFS-MCS; CFS-FM; or CFS-MCS-FM

Outcome Measure	CFS alone (n = 50)	CFS-MCS (n = 27)	CFS-FM (n = 18)	CFS-MCS-FM (n = 19)
Mean (Standard Deviation)				
MOS-SF-36				
Physical Functioning	53.61 (25.05)^a^	42.50 (19.66)	40.56 (23.69)	32.89 (19.95)^a^
Role-Physical	4.69 (12.27)	3.85 (11.60)	4.17 (12.86)	3.95 (12.54)
Bodily Pain	49.83 (21.88)^a,*c*^	40.62 (21.63)^b^	30.00 (13.18)^c^	21.58 (18.60)^a, b^
General Health	36.65 (16.74)^a^	28.75 (15.94)	38.54 (16.62)^b^	19.95 (32.24)^a, b^
Vitality	20.73 (15.98)	15.19 (12.84)	19.72 (16.31)	11.32 (9.84)
Social Functioning	46.09 (25.15)^a^	30.29 (21.84)^a^	47.22 (23.70)	30.26 (19.68)
Role-Emotional	56.74 (45.00)	47.44 (45.39)	42.59 (35.80)	57.89 (41.34)
Mental Health	67.08 (16.30)	55.85 (19.35)	66.22 (14.79)	62.11 (18.10)
Brief COPE				
Self Distraction	2.30 (0.87)^a, b^	2.91 (0.81)^b^	2.89 (0.88)	3.18 (0.65)^a^
Active Coping	2.97 (0.84)	3.15 (0.83)	3.28 (0.73)	3.10 (0.88)
Denial	1.41 (0.85)	1.28 (0.59)	1.25 (0.35)	1.32 (0.61)
Substance Use	1.15 (0.47)	1.22 (0.63)	1.53 (0.98)	1.24 (0.42)
Emotional Support	2.40 (0.95)	2.17 (0.91)	2.56 (0.97)	2.29 (0.85)
Instrumental Support	2.32 (0.96)	2.61 (0.93)	2.78 (0.75)	2.66 (0.99)
Behavioral Disengagement	1.33 (0.51)	1.27 (0.38)	1.47 (0.44)	1.47 (0.94)
Venting	1.93 (0.63)	2.09 (0.77)	2.39 (0.65)	2.11 (.077)
Positive Reframing	2.20 (1.06)	2.28 (0.94)	2.75 (0.96)	2.53 (1.05)
Planning	2.97 (0.97)	3.00 (0.98)	3.00 (0.86)	3.03 (0.86)
Humor	1.68 (0.79)^a^	1.78 (0.87)	2.39 (1.20)^a^	1.79 (0.77)
Acceptance	2.95 (0.88)	2.81 (0.83)	2.94 (0.75)	2.95 (0.80)
Religion	2.41 (1.01)	2.44 (0.97)	2.31 (1.06)	2.47 (1.05)
Self Blame	1.69 (0.87)	1.74 (0.75)	2.00 (0.77)	1.92 (0.79)
Beck Depression Inventory	16.47 (8.61)^a^	20.88 (9.64)	18.50 (9.54)	23.93 (11.43)^a^
Fatigue Severity Scale	5.98 (0.68)	6.35 (0.66)^a^	5.70 (1.22)^a^	6.30 (0.53)
Pittsburgh Sleep Quality Index	7.26 (2.53)^a^	8.69 (2.20)	8.94 (2.10)	8.95 (2.39)^a^
Brief Pain Inventory				
Interference	2.83 (2.52)^a, c,*d*^	4.56 (2.78)^b, c^	5.36 (1.96)^d^	6.80 (2.33)^a, b^
Severity	3.03 (2.06)^a, c^	4.06 (2.02)^b^	4.86 (1.40)^c^	6.10 (2.04)^a, b^
Symptoms				
Sore Throat	23.50 (26.61)	29.48 (26.97)	20.56 (26.40)	24.84 (28.82)
Tender Lymph Nodes	25.75 (30.36)	23.40 (26.33)	27.50 (33.79)	32.00 (27.92)
Muscle Pain	50.52 (28.34)^a, b^	60.76 (28.22)	72.92 (22.56)^b^	78.42 (24.97)^a^
Joint Pain	31.85 (33.25)^a, c^	46.04 (35.95)^b^	63.06 (32.18)^c^	75.50 (24.54)^a, b^
Impaired Memory	62.96 (25.22)	59.63 (23.82)	65.14 (25.53)	68.94 (22.65)
Unrefreshing Sleep	76.89 (18.89)	75.19 (25.43)	89.67 (10.86)	87.03 (14.46)
Post-Exertional Malaise	73.27 (17.84)	75.19 (19.16)	74.86 (23.74)	82.36 (14.10)
Headaches	42.91 (33.39)^a^	56.87 (30.50)	55.00 (31.44)	74.03 (24.39)^a^
Six Minute Walk Test				
Distance Walked (feet)	1419.47 (312.17)	1728.08 (2387.62)	1304.28 (356.492)	1221.39 (239.23)
Average RPE	9.85 (2.24)^a^	10.56 (2.08)	11.37 (2.76)	11.78 (2.42)^a^
Sit and Reach (inches)	13.48 (3.54)	13.16 (4.49)	14.55 (4.24)	13.40 (3.77)
Hand Grip (pounds)				
Right	64.45 (24.67)	61.57 (21.21)	64.19 (21.87)	52.28 (15.34)
Left	60.04 (22.71)	58.63 (17.90)	58.72 (20.71)	48.07 (17.21)
Actigraphy Mean	161.35 (58.55)	145.74 (58.13)	167.23 (48.51)	149.78 (63.12)
Lifetime Axis I Diagnosis %	21 (42.0%)	10 (37.0%)	6 (33.3%)	6 (31.6%)
Current Axis I Diagnosis %	17 (34.0%)	5 (18.5%)	7 (38.9%)	6 (31.6%)

Similar letters across diagnostic groups significantly differ at the p < .05 level.

An ANOVA was conducted to compare individuals on the Beck Depression Inventory. There was a significant difference in depression scores overall (F (3,107) = 3.20, p < .05). Post hoc tests revealed that individuals with CFS-MCS-FM experienced significantly more depression than individuals with CFS alone (see Table 1). However, χ^2 ^analyses did not yield significant differences in current psychiatric diagnoses (χ^2 ^(3, N = 114) = 2.71, p=.44) or lifetime psychiatric diagnoses (χ^2 ^(3, N = 114) = .85, p=.84) across the four groups.

Findings from ANOVAs also showed significant overall differences in sleep quality (F (3,106) = 4.08, P < .01), and fatigue severity (F (3,107) = 3.20, p < .05). Post hoc analyses revealed that individuals with CFS-MCS-FM experienced significantly more sleep problems than individuals with CFS alone. Individuals with CFS-MCS were found to have significantly higher fatigue severity than individuals with CFS-FM (see Table 1).

The Brief Pain Inventory examined pain severity and pain interference with functioning. ANOVAs revealed overall significant differences between the four groups on pain severity, F (3,104) = 11.65, p < .001. For this dimension, individuals with CFS-MCS-FM experienced significantly higher pain severity than individuals with CFS alone and individuals with CFS-MCS. Additionally, individuals with CFS-FM had significantly more pain severity than individuals with CFS alone. The interference with functioning dimension also revealed overall significant differences, F (3, 103) = 12.44, p < .001. Post hoc analyses revealed that individuals with CFS-MCS-FM had significantly more interference in functioning due to pain than individuals with CFS alone and individuals with CFS-MCS. Both individuals with CFS-MCS and individuals with CFS-FM had significantly more interference in functioning due to pain than individuals with CFS alone (see Table 1).

For the scales measuring coping, two revealed overall significant differences, including self distraction (F (3,106) = 6.59, p < .001) and humor (F (3,107) = 2.90, p < .05). Individuals with CFS-MCS, and individuals with CFS-MCS-FM used significantly more self distraction than individuals with CFS alone. Individuals with CFS-FM used significantly more humor as a coping style than individuals with CFS alone (see Table 1).

ANOVAs were also conducted for severity of the eight core CFS symptoms. There were significant group differences for muscle pain (F (3,109) = 6.19, p < .001), joint pain (F (3,110) = 9.96, p < .001), unrefreshing sleep (F (3,107) = 3.33, p < .05), and headaches (F (3,108) = 4.62, p < .01). Table 1 shows the group differences based on post hoc analyses. The CFS-MCS-FM group reported significantly more severe muscle pain, joint pain, and headaches than the CFS alone group. They also reported significantly more joint pain severity than the CFS-MCS group. The CFS-FM group reported significantly higher severity of muscle pain and joint pain than the CFS alone group.

No significant differences were found across diagnostic groups for physical measures including the six minute walk test, sit and reach, hand grip strength, and actigraphy. Outcomes for physical measures are reported in Table 1. During the six minute walk test, RPE was calculated every 30 seconds. Mean RPE scores were calculated, and overall significant differences were found between groups (F (3, 102) = 3.65, p < .05). Post hoc analyses revealed that the CFS alone group had a significantly lower RPE score than the CFS-MCS-FM group.

## Discussion

These results suggest that a substantial number (56%) of patients with CFS also meet criteria for MCS, FM, or both. These findings are consistent with previous research indicating high rates of overlap among these illnesses [11,23]. While some argue that distinguishing between these three syndromes is not merited due to high rates of co-occurrence and overlapping symptom criteria [55], we found a general pattern indicating that individuals with all three diagnoses experience more overall difficulties when compared to individuals with two or fewer diagnoses. Participants with CFS-MCS-FM experienced more depression and poorer sleep quality than those with CFS alone. They also had poorer physical functioning, more bodily pain, and poorer general health than those with CFS alone. Those with all three diagnoses also had lower general health scores than those with CFS-FM, and more bodily pain than those with CFS-MCS.

In addition, the CFS-MCS group had lower social functioning scores on the SF-36 than those with CFS alone. Our findings regarding the two groups that included individuals with MCS (CFS- MCS and CFS-MCS-FM) are consistent with previous research suggesting that individuals with MCS tend to have low scores on the SF-36 health status scales [56]. Of note, the two MCS groups also utilized self-distraction as a coping mechanism more than the CFS alone, as measured by the Brief Cope. Self-distraction is considered a maladaptive coping style on this measure [57]. It has been hypothesized that individuals with MCS may consider chemical exposure to be unavoidable, leading them to utilize passive coping [58]. Lower social functioning of these individuals and a passive coping style may be a consequence of not being able to avoid chemical exposure.

Although pain is a symptom associated with CFS, it is the hallmark symptom of FM. The two groups that included individuals with FM (CFS-FM and CFS-MCS-FM) reported more bodily pain on the SF-36 than the CFS alone and CFS-MCS groups. Individuals with FM also reported more pain severity and pain interference than the non-FM groups. Additionally, among the eight core symptoms of CFS, they reported more severe headaches, muscle pain, and joint pain than the other groups. This suggests that although many symptoms relating to pain overlap among the disorders, pain-related symptoms are more salient among patients with FM.

High rates of psychiatric comorbidity have been found in studies of patients with CFS, MCS, and FM [59]. Our sample had rates of current psychiatric diagnosis ranging from 18.5% for the CFS-MCS group to 38.9% for the CFS-FM group. In contrast to Ciccone and Natelson [23], we did not find significant differences between groups with regards to psychiatric illness. However, the CFS-MCS-FM group had the highest depression scores, suggesting that depression may be more prevalent in individuals who are living with more severe disability.

We did not find differences between groups for the in vivo physical measures of strength, exercise tolerance, and flexibility. This is in contrast to the various differences found on self-report measures related to physical functioning and disability. Of note, the CFS-MCS-FM group rated a significantly higher level of exertion on the six minute walk test than the CFS alone group. It is possible that the six minute walk test was not strenuous enough to detect differences in performance across groups, but RPE scores suggest that the task was more difficult for the CFS-MCS-FM group. Future research might examine these differences during a higher-intensity activity.

## Conclusion

It is important to note that all participants in this study met diagnostic criteria for CFS; so many symptoms were expected to be present across all participants. Individuals with CFS alone were the highest functioning group across several domains, such as disability, depression, and severity of symptoms. In contrast, participants with CFS-MCS-FM experienced the greatest amount of overall disability. However, physical measures of disability were not consistent with these findings, as there were no differences across groups. Individuals with all three diagnoses found the six minute walk test to be more intense, as measured by RPE, compared to the CFS alone group. This study provides evidence that having more than one diagnosis exacerbates one's disability above and beyond CFS alone.

## Competing interests

The author(s) declare that they have no competing interests.

## Authors' contributions

MB and LJ both conceived of the study. MB carried out the statistical analyses and contributed to the writing of the manuscript. LJ participated in the design of the study and contributed to the writing of the manuscript. All authors read and approved the final manuscript.
