# Functioning in individuals with chronic fatigue syndrome: increased impairment with co-occurring multiple chemical sensitivity and fibromyalgia

**DOI:** 10.1186/1476-5918-6-9

**Published:** 2007-07-30

**Authors:** Molly M Brown, Leonard A Jason

**Affiliations:** 1 Department of Psychology, DePaul University, Center for Community Research, Chicago, IL, USA

## Correction

In our publication [1], the frequencies and percentages of lifetime and current Axis I disorders reported in Table 1 were found to be inaccurate. The values reported in Table 1 of this version have been corrected. All other outcome variables in Table 1 were reported correctly in the original version of the article. The analyses and results in the text were accurate in the original version of the paper.

**Table 1:** Outcome measures for individuals with CFS alone; CFS-MCS; CFS-FM; or CFS-MCS-FM

Outcome Measure	CFS alone (n = 50)	CFS-MCS (n = 27)	CFS-FM (n = 18)	CFS-MCS-FM (n = 19)
Mean (Standard Deviation)				
MOS-SF-36				
Physical Functioning	53.61 (25.05)^a^	42.50 (19.66)	40.56 (23.69)	32.89 (19.95)^a^
Role-Physical	4.69 (12.27)	3.85 (11.60)	4.17 (12.86)	3.95 (12.54)
Bodily Pain	49.83 (21.88)^a,c^	40.62 (21.63)^b^	30.00 (13.18)^c^	21.58 (18.60)^a,b^
General Health	36.65 (16.74)^a^	28.75 (15.94)	38.54 (16.62)^b^	19.95 (32.24)^a,b^
Vitality	20.73 (15.98)	15.19 (12.84)	19.72 (16.31)	11.32 (9.84)
Social Functioning	46.09 (25.15)^a^	30.29 (21.84)^a^	47.22 (23.70)	30.26 (19.68)
Role-Emotional	56.74 (45.00)	47.44 (45.39)	42.59 (35.80)	57.89 (41.34)
Mental Health	67.08 (16.30)	55.85 (19.35)	66.22 (14.79)	62.11 (18.10)
Brief COPE				
Self Distraction	2.30 (0.87)^a,b^	2.91 (0.81)^b^	2.89 (0.88)	3.18 (0.65)^a^
Active Coping	2.97 (0.84)	3.15 (0.83)	3.28 (0.73)	3.10 (0.88)
Denial	1.41 (0.85)	1.28 (0.59)	1.25 (0.35)	1.32 (0.61)
Substance Use	1.15 (0.47)	1.22 (0.63)	1.53 (0.98)	1.24 (0.42)
Emotional Support	2.40 (0.95)	2.17 (0.91)	2.56 (0.97)	2.29 (0.85)
Instrumental Support	2.32 (0.96)	2.61 (0.93)	2.78 (0.75)	2.66 (0.99)
Behavioral Disengagement	1.33 (0.51)	1.27 (0.38)	1.47 (0.44)	1.47 (0.94)
Venting	1.93 (0.63)	2.09 (0.77)	2.39 (0.65)	2.11 (.077)
Positive Reframing	2.20 (1.06)	2.28 (0.94)	2.75 (0.96)	2.53 (1.05)
Planning	2.97 (0.97)	3.00 (0.98)	3.00 (0.86)	3.03 (0.86)
Humor	1.68 (0.79)^a^	1.78 (0.87)	2.39 (1.20)^a^	1.79 (0.77)
Acceptance	2.95 (0.88)	2.81 (0.83)	2.94 (0.75)	2.95 (0.80)
Religion	2.41 (1.01)	2.44 (0.97)	2.31 (1.06)	2.47 (1.05)
Self Blame	1.69 (0.87)	1.74 (0.75)	2.00 (0.77)	1.92 (0.79)
Beck Depression Inventory	16.47 (8.61)^a^	20.88 (9.64)	18.50 (9.54)	23.93 (11.43)^a^
Fatigue Severity Scale	5.98 (0.68)	6.35 (0.66)^a^	5.70 (1.22)^a^	6.30 (0.53)
Pittsburgh Sleep Quality Index	7.26 (2.53)^a^	8.69 (2.20)	8.94 (2.10)	8.95 (2.39)^a^
Brief Pain Inventory				
Interference	2.83 (2.52)^a,c,d^	4.56 (2.78)^b,c^	5.36 (1.96)^d^	6.80 (2.33)^a,b^
Severity	3.03 (2.06)^a,c^	4.06 (2.02)^b^	4.86 (1.40)^c^	6.10 (2.04)^a,b^
Symptoms				
Sore Throat	23.50 (26.61)	29.48 (26.97)	20.56 (26.40)	24.84 (28.82)
Tender Lymph Nodes	25.75 (30.36)	23.40 (26.33)	27.50 (33.79)	32.00 (27.92)
Muscle Pain	50.52 (28.34)^a,b^	60.76 (28.22)	72.92 (22.56)^b^	78.42 (24.97)^a^
Joint Pain	31.85 (33.25)^a,c^	46.04 (35.95)^b^	63.06 (32.18)^c^	75.50 (24.54)^a,b^
Impaired Memory	62.96 (25.22)	59.63 (23.82)	65.14 (25.53)	68.94 (22.65)
Unrefreshing Sleep	76.89 (18.89)	75.19 (25.43)	89.67 (10.86)	87.03 (14.46)
Post-Exertional Malaise	73.27 (17.84)	75.19 (19.16)	74.86 (23.74)	82.36 (14.10)
Headaches	42.91 (33.39)^a^	56.87 (30.50)	55.00 (31.44)	74.03 (24.39)^a^
Six Minute Walk Test				
Distance Walked (feet)	1419.47 (312.17)	1728.08 (2387.62)	1304.28 (356.492)	1221.39 (239.23)
Average RPE	9.85 (2.24)^a^	10.56 (2.08)	11.37 (2.76)	11.78 (2.42)^a^
Sit and Reach (inches)	13.48 (3.54)	13.16 (4.49)	14.55 (4.24)	13.40 (3.77)
Hand Grip (pounds)				
Right	64.45 (24.67)	61.57 (21.21)	64.19 (21.87)	52.28 (15.34)
Left	60.04 (22.71)	58.63 (17.90)	58.72 (20.71)	48.07 (17.21)
Actigraphy Mean	161.35 (58.55)	145.74 (58.13)	167.23 (48.51)	149.78 (63.12)
Lifetime Axis I Diagnosis %	29 (58.0%)	17 (63.0%)	12 (66.7%)	13 (68.4%)
Current Axis I Diagnosis %	19 (38.0%)	8 (29.6%)	9 (50.0%)	8 (42.1%)

Similar letters across diagnostic groups significantly differ at the p < .05 level.
